# Supplementary material for: Construction of Candida albicans Adhesin-Exposed Synthetic Cells for Preventing Systemic Fungal Infection
Source: Vaccines (Basel). 2023 Sep 25;11(10):1521. doi: 10.3390/vaccines11101521 (PMC10611093; doi:10.3390/vaccines11101521)
Supplement: Supplementary file 1 [file vaccines-11-01521-s001.zip › vaccines-2596493-supplementary.pdf]

Construction of *Candida albicans* adhesin-exposed synthetic cells for preventing systemic fungal infection

Zirun Zhao [1], Ying Sun [1], Mingchun Li [1], and Qilin Yu [1,2,3]*

[1] Key Laboratory of Molecular Microbiology and Technology, Ministry of Education, Department of Microbiology, College of Life Sciences, Nankai University, Tianjin 300071, China; 2120221390@mail.nankai.edu.cn (Z.Z.); 2120231588@mail.nankai.edu.cn (Y.S.); nklimingchun@163.com (M.L.); yuqilin@mail.nankai.edu.cn (Q.Y.)
[2] Research Center for Infectious Diseases, Nankai University, Tianjin, 300350, China; yuqilin@mail.nankai.edu.cn (Q.Y.)
[3] National Key Laboratory of Intelligent Tracking and Forecasting for Infectious Diseases, Tianjin, 300350, China; yuqilin@mail.nankai.edu.cn (Q.Y.)

* Correspondence: yuqilin@mail.nankai.edu.cn

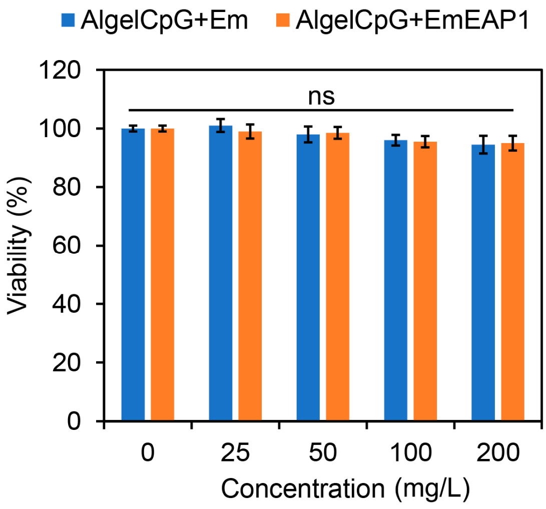

Figure S1. Effect of the vaccines on the viability of the DC2.4 dendritic cells.

Citation: To be added by editorial staff during production.

Academic Editor: Firstname Lastname

Received: date
Revised: date
Accepted: date
Published: date

Copyright: © 2023 by the authors. Submitted for possible open access publication under the terms and conditions of the Creative Commons Attribution (CC BY) license (https://creativecommons.org/licenses/by/4.0/).
